# Does Patient Compliance Influence Wearable Cardioverter Defibrillator Effectiveness? A Single-Center Experience

**DOI:** 10.3390/jcm12144743

**Published:** 2023-07-18

**Authors:** Luca Fazzini, Maria Francesca Marchetti, Ferdinando Perra, Mattia Biddau, Nicola Massazza, Vincenzo Nissardi, Elena Agus, Roberta Demelas, Roberta Montisci

**Affiliations:** Clinical Cardiology Unit, Department of Medical Sciences and Public Health, University Hospital of Cagliari, Via Carrara 25, 09125 Cagliari, Italy; mfrancescacardio@gmail.com (M.F.M.); ferdinando.perra@gmail.com (F.P.); mattiabiddau@gmail.com (M.B.); nicolamassazza@gmail.com (N.M.); vinissardi@tiscali.it (V.N.); elenaagus@hotmail.com (E.A.); roberta.demelas@hotmail.it (R.D.); rmontisc@gmail.com (R.M.)

**Keywords:** sudden cardiac death, acute coronary syndrome, heart failure, HFrEF, wearable cardioverter defibrillator

## Abstract

The study was designed to assess patient adherence to wearable cardioverter defibrillator as an indicator of device effectiveness. The patient training is not widely properly standardized. We enrolled 25 patients with a wearable cardioverter defibrillator to prevent sudden cardiac death between June 2020 and August 2022. Among them, 84% were male with a median age of 63.6 years. The indication was an ischemic (44%) and a non-ischemic (56%) disease. The patients were followed-up until the decision to upgrade to an implantable device was taken. We trained the patients according to our suggested protocol. The median wear time was 90 days, and the median daily wear time was 23.5 h, similar throughout sex, age, and indication groups. In total, 24% of the participants underwent cardioverter defibrillator implantation. Between the device-implanted and non-implanted groups, left ventricular ejection fraction and left ventricular indexed end-diastolic volume were significantly different (EF 35.8 ± 12 vs. 46.4 ± 8.5%, *p* = 0.028, iEDV 108 ± 52 vs. 70.7 ± 21.1 mL/m^2^, *p* = 0.024). We did not find any differences in cardiac magnetic resonance data, even though all patients who underwent device implantation had late gadolinium enhancement spots. Our results support standardized patient training to obtain great patient adherence to the instructions to the wearable device and therefore its effectiveness.

## 1. Introduction

Sudden cardiac death (SCD) is a potentially fatal event if not treated in time with external interventions such as cardio-pulmonary resuscitation and defibrillation. Nowadays, the use of the wearable cardioverter defibrillator (WCD) can lead to the availability of different approaches in SCD prevention. The WCD may be indicated as a bridge to recovery by implantable cardioverter defibrillator (ICD) implantation or by helping to make an important clinical decision.

The WCD protects against SCD during the immediate phase after re-perfused myocardial infarction or 90 days after coronary revascularization without acute coronary syndrome even if the literature is controversial [1] or in patients with a recent finding of non-ischemic myocardial dysfunction and potentially reversible as takotsubo syndrome [2,3]. After that time frame, ICD implantation may be indicated. Indeed, according to some studies, ICD implanted immediately after myocardial infarction did not show long-term mortality benefits [4] even though it is clear that the ICD reduces mortality among patients with a reduced ejection fraction when the device is implanted months to years after myocardial infarction [5,6,7]. The WCD is a device that allows the clinician to have time to make the best decision while the patient is protected against SCD, and meanwhile offers time for non-antiarrhythmic drugs to act on SCD risk reduction [8,9].

Effectiveness of the WCD has been recognized and defibrillation of VF has been demonstrated, but randomized clinical trials and studies which show long-term mortality benefit are still lacking. Patient compliance to follow all the given rules plays a pivotal role in proving that the WCD is effective in detecting arrythmias and the need for intervention. Lacking in patient responsibility leads, unavoidably, to a downfall in the effectiveness of a WCD.

The wearable cardioverter defibrillator.

The WCD is a vestlike device intended to be worn 24 h per day underneath clothing except while taking a shower. It automatically delivers an electric shock when ventricular fibrillation (VF) or ventricular tachycardia (VT) are detected. Before the electric shock is delivered, the patient is warned by receiving vibrations and loud alarms in order to allow the patient to stop the shock themself if they are conscious, avoiding inappropriate therapy.

The device is provided with batteries which can work continuously for 24 h. Battery level is monitored by a software which warns the patient if it has to be recharged or replaced.

The WCD indications, which have been well documented in a recent position paper [10], include patients with a high risk of SCD in the context of recent myocardial infarction, recent revascularization (since ICD is contraindicated in the forty days after myocardial infarction and three months after revascularization in ischemic cardiomyopathy), non-ischemic cardiomyopathy, and myocarditis. Moreover, it is appropriate considering WCD in patients with temporary removed ICD, most commonly due to infection, as a bridge to new ICD implantation.

## 2. Materials and Methods

### 2.1. Aim

Our study aimed to assess patient adherence to WCD wearing, to evaluate WCD efficacy and safety in VT/VF recognition and treatment in a high-risk SCD population, and to quantify the number of patients which implanted ICD after the WCD-wearing timeframe recommended according to the 2015 Sudden Cardiac Death Prevention ESC guidelines [11].

### 2.2. Participants

We enrolled 25 patients which underwent WCD application between June 2020 and August 2022 in the Clinal Cardiology Unit of the “Policlinico Universitario di Monserrato”, University of Cagliari. They received the WCD in order to prevent the SCD while waiting for left ventricle ejection fraction recovery or better definition of the ventricular dysfunction etiology according to the 2015 Sudden Cardiac Death Prevention ESC guidelines and the 2021 Heart Failure ESC guidelines [11,12].

Among the patients, 21 were male (84%) with a median age of 63.6 ± 14.6 (range 21–86). Baseline patient characteristics are reported in Table 1.

**Figure 1:**
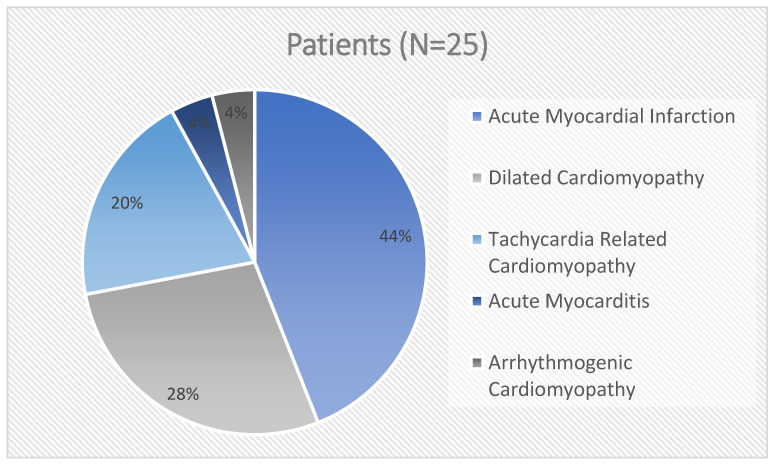
Wearable cardioverter defibrillator indications.

The WCD indication was an ischemic disease in 11 patients (44%) and a non-ischemic disease in 14 patients (56%). Among the non-ischemic disease group, we reported 7 (28%) idiopathic dilated cardiomyopathies, 5 (20%) tachi-induced cardiomyopathies, 1 (4%) suspected arrhythmogenic cardiomyopathy and 1 (4%) arrhythmic presentation myocarditis (Figure 1).

### 2.3. Study Design and Methods

We enrolled the patients who received a WCD in our Clinical Cardiology Unit following them up until the decision to implant or not implant an ICD was made. At the time of WCD application, we collected clinical, electrocardiogram, echocardiogram, and pharmacological treatment data. We performed echocardiogram during the follow up until WCD was removed. The echo examinations aimed to evaluate left ventricular dimensions, volumes, systolic and diastolic function, wall motion score index, and right ventricular function.

Some of the patients (64%) underwent cardiac magnetic resonance (CMR) assessing CINE, T1, T2 sequences and after gadolinium infusion for late gadolinium enhancement. Our CMR imaging protocol is composed of short and long axis steady-state free precession (SSFP) in T2-STIR sequences, T2-mapping and late gadolinium enhancement. According to SSPF sequences and the Simpson method, we collected the following parameters: end-diastolic volumes, end-systolic volumes, stroke volume, left and right ventricle ejection fraction, 17 segments kinesis, left and right atrium area.

The myocardial tissue characterization was reached with T2-STIR and T2 mapping before the contrast medium administration. Our range T2 mapping values were 53 ± 3 ms.

Finally, the late gadolinium enhancement was evaluated after a 10–12 min paramagnetic contrast medium infusion (Gadovist, Bayer Healthcare, Berlin, Germany) through phase-sensitive inversion recovery (PSIR) sequences.

The WCD setting was adjusted based on patient characteristics and previous arrhythmias. Most of them were set at 150 bpm for VT identification and 60 s to shock, 200 bpm for VF identification and 25 s to shock. We raised up the VT threshold in some of the youngest and more physically active patients. All the patients were followed up at 1 and 3 months. Some of them were followed up after more than 3 months depending on specific clinical scenarios.

### 2.4. How We Train the Patient

**Figure 2:**
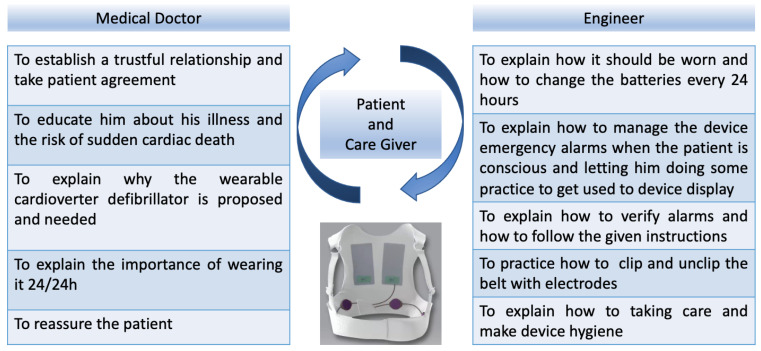
The education of the patient plays a vital role in wearable cardioverter defibrillator effectiveness. Herein we provide the steps we suggest following in order to have a coordinate education program.

Before providing the patient with a WCD, the medical doctor and the engineer from the parent company have a crucial role in training the patient, teaching them different important tasks (Figure 2). The medical doctor carries out counseling with the patient and the caregiver when possible and available to explain why the WCD is being proposed, what it is used for, how it works, the importance of wearing it 24/24 h except for the moments when the patient takes a shower and tries to reassure the patient. The medical doctor training sessions are conducted at the hospital bed, day by day, aiming to raise awareness about the relevance of the daily wear time to achieve effectiveness.

Once the patient is eligible, shortly before being discharged from the clinic, they undergo training with the engineer who educates the patient in a dedicated and quiet environment. The training session usually lasts for about one hour and is composed of various and sequential steps. The WCD operation is briefly described, and the engineer provides training about the main skills which the patient must learn during the training session such as highlighting, again, the importance of wearing it 24/24 h, to change batteries once per day, to manage the alarms, to follow the instructions on the display and press the answer keys.

All the steps are illustrated by the engineer and then the patient and the caregiver repeat all the steps with the help and supervision of the engineer until the patient is autonomous. The patient is then given a leaflet summarizing all the instructions and given a toll-free number to contact at any time.

### 2.5. Statistical Analysis

Continuous variables are expressed as mean ± standard deviation or median and categorical data as percentages. Continuous variables were analyzed with Student’s *t* test for paired and unpaired data and the Mann–Whitney test for non-parametric data. The chi-square test was used for the analysis of categorical variables. A *p*-value < 0.05 was considered significant. Data were analyzed with SPSS software, version 22.0.

## 3. Results

The adherence was defined as the medium wear time measured by Zoll^®^LifeVest^®^ software (Version V07.9). The median wear time was 90 days (CI 41–146 days), and the median daily wear time was 23.5 h (CI 10.5–23.99 h). All patients were adherent to the treatment except for two who wore the WCD discontinuously with median daily wear time of 10.5 and 14.48 h.

The median daily wear time was similar between sex, age, indication and total wear days groups.

Two different medical doctor investigators assessed arrhythmic events during the WCD utilization through the Zoll^®^LifeVest^®^ net database. The arrhythmic events were classified according to the 2015 Sudden Cardiac Death Prevention ESC guidelines 11: sustained VT (>30 s) or VF treated with appropriate shock; sustained VT with aborted shock by pressing the patient response buttons during the alarm; inappropriate shock (without VT/VF); non-sustained VT (three or more ventricular beats spontaneously ended within 30 s); inappropriate alarm without VT/VF.

We observed a sustained VT properly treated with appropriate shock in a patient discharged 9 days earlier with anterior myocardial infarction treated with primary angioplasty and reduced left ventricular ejection fraction (33%). No deaths were reported. We reported 91 inappropriate arrhythmic alarms due to artifacts without any inappropriate shock. Among 25 patients included in our study, 6 (24%) underwent ICD implantation (three ischemic cardiomyopathies and three non-ischemic cardiomyopathies). Among the implanted patients, five underwent ICD implantation as they did not recover in left ventricle ejection fraction after optimal medical therapy, and arrhythmogenic cardiomyopathy diagnosis with desmoplakin mutation was assigned in the sixth patient.

We found a significative difference at follow-up between the ICD implanted and non-implanted groups in terms of left ventricular ejection fraction and left ventricular indexed end-diastolic volume, which were reduced and increased, respectively (EF 35.8 ± 12 vs. 46.4 ± 8.5%, *p* = 0.028, iEDV 108 ± 52 vs. 70.7 ± 21.1 mL/m^2^, *p* = 0.024) (Table 2). We did not find gender differences among the groups.

We did not find any significant differences in CMR findings between the two groups, even if all patients who underwent ICD implantation had late gadolinium enhancement spots.

## 4. Discussion

A wearable cardioverter–defibrillator is a temporary treatment option for patients at high risk for SCD and for patients who are temporarily not candidates for an implantable cardioverter defibrillator. Our single-center study aimed to evaluate adherence, efficacy, and safety of WCD in a high-risk SCD population.

Our data show that an efficient patient education and training to handle the WCD leads to an excellent patient adherence to wearing and managing the device. This has been demonstrated by the elevated wear time throughout all the treatment time, which is even higher than that of the previous largest randomized trial [1] and comparable to the world best median daily wear time in the WEARIT-FRANCE study [13]. More specifically, the VEST trial enrolled patients in 2008. At that time, the WCD was delivered to the patient without any structured education and this fact surely affected the negative trial’s primary outcome. Moreover, most of the sudden cardiac deaths in the device group occurred when the patient was not wearing the device or due to arrhythmic storm. Such an analysis strengthens the central role of patient education, allowing us to underline how important the patient training and their involvement are in order to achieve a safe and effective treatment. Patient training is the cornerstone of WCD efficacy.

In addition, in our population, all the malignant arrhythmias have been recognized and effectively treated; therefore, it is as safe and effective as expected. Our data are comparable with the results of previous studies which demonstrated efficacy and safety of the WCD in different clinical settings [14,15,16,17,18,19,20]. We reported a 100% efficacy in interrupting malignant arrhythmias with a single shock (up to a 98% efficacy in the literature) [21].

Even the inappropriate alarms were less common than expected considering the long monitoring time; the WCD Swedish Registry reported 91% of inappropriate alarms, which were more frequent in the obese patients. We did not find any linkage between body mass index and inappropriate alarms [22].

Given the small sample size, it is important to highlight the two enrolled patients who wore the WCD discontinuously with a median daily wear time of 10.5 and 14.48 h and understand the non-compliance issues. Both were non-compliant to medical therapy even before WCD delivery, and such an experience suggests that patient compliance to medical therapy may guide the clinician to a proper selection for WCD.

At the end of the follow-up, only 24% of the patients had an ICD indication. It means that 76% did not have an ICD indication anymore. This result, comparable with the literature, supports the use of the WCD to more accurately select the patient who needs an ICD and avoid unnecessary implantations. Even if our study did not include an economic analysis, the widespread WCD utilization could reduce the healthcare expenditure, as some studies already demonstrated, considering that three out of four patients with WCD do not have ICD indication at the end of the recommended follow-up [23].

Among the 25 patients included, 16 underwent CMR, but we did not find any significative differences between the two groups. All the patients with ICD indication at the end of the follow-up have late gadolinium enhancement spots at the late acquired CMR sequences. It is well known that late gadolinium enhancement and fibrotic scars correlate with adverse cardiovascular events and mortality in patients with dilated cardiomyopathy and reduced ejection fraction [24,25] and, recently, in patients with dilated non-ischemic cardiomyopathy with mildly reduced ejection fraction [26,27].

It would be desirable to have clinical and imaging parameters, more accurate than ejection fraction, to better stratify SCD risk in this population, and certainly CMR has a promising role. According to our results, the ICD-implanted group had a significantly higher left ventricular indexed end-diastolic volume (*p* = 0.02); such a finding allows consideration of this echocardiographic parameter, among left ventricular ejection fraction, arrhythmias and late gadolinium enhancement spots, to properly select the patients who might benefit the most from the WCD.

The research is ongoing in this field, aiming to describe a new approach to SCD risk stratification, away from the simple dichotomy approach based on the ejection fraction towards a personalized assessment of individual risk [28].

On the one hand, the arrhythmic SCD is an avoidable death, and the Zoll^®^LifeVest^®^ could be a safe and effective option in patients who are not candidates for ICD yet [29].

We highlighted the importance of standardized patient training to achieve high adherence and consequently improve the effectiveness of the wearable device. We suggest to implement and standardize such a training protocol in accordance with all the ward medical doctors who may indicate the device and the referring engineer. Defining a protocol leads to a better definition of medical doctor and engineer duties and gives relevance raising awareness about patient training which is pivotal in the effectiveness of this wearable device.

Our study has some limitations. First, it was a single-center retrospective and non-randomized study with a small patient sample. Specifically, the small sample size surely affected the differences between the ICD-implanted and non-implanted groups. However, our results in terms of wear time and effectiveness are comparable to those of the larger published registries. Second, the decision to implant an ICD or to wear a WCD was made by the clinician in a non-standardized way, so it could have led to biased patient selection.

## 5. Conclusions

We evaluated patient adherence, efficacy, and safety of the wearable cardioverter defibrillator in a high-risk sudden cardiac death population. In our single-center retrospective study, we demonstrated how important it is to carefully coordinate patient training, leading to a greater probability of patient adherence to leaving on the wearable cardioverter defibrillator. Moreover, we confirmed device efficacy and safety, which are closely related to patient adherence.

## Figures and Tables

**Table 1:** Baseline characteristics.

Baseline Characteristics	Patients (N = 25)
Age (year)_Mean ± SD	63.6 ± 14.6
Female sex	16%
BMI (kg/m^2^)_Mean ± SD	26.0 ± 5.8
Hypertension	56%
Dyslipidemia	64%
Diabetes	36%
Tobacco use	28%
Coronary artery disease	52%
Right bundle branch block	16%
Left bundle branch block	12%
Dual antiplatelets therapy	24%
ACEi	32%
ARB	64%
MRA	68%
Beta blockers	96%
Loop diuretics	72%

**Table 2:** Clinical and echocardiographic characteristics of patients with and without an ICD implant.

Follow-Up Analysis	ICD Not Implanted Group (19)	ICD Implanted Group (6)	*p*-Value
Age_year (Mean (SD))	63.8 (15.1)	63.1 (14.3)	0.93
Sex_males (N (%))	15 (79%)	6 (100%)	0.54
BMI_kg/m^2^ (Mean (SD))	25.7 (5.8)	27.1 (6.7)	0.67
**LVEF Simpson_% (Mean (SD))**	46.4 (8.5)	35.8 (12.8)	**0.028**
Left ventricular end-diastolic dimension_mm (Mean (SD))	59.7 (9.4)	55.0 (8.3)	0.27
Left ventricular end-diastolic volume_mL (Mean (SD))	132.5 (52.8)	123.5 (113.9)	0.85
**Left ventricular indexed end-diastolic** **volume_mL/m^2^ (Mean (SD))**	70.7 (21.2)	108.0 (52.2)	**0.024**

## Data Availability

The data presented in this study are already available in this “article”.
